# Pupal Diapause Termination and Transcriptional Response of *Antheraea pernyi* (Lepidoptera: Saturniidae) Triggered by 20-Hydroxyecdysone

**DOI:** 10.3389/fphys.2022.888643

**Published:** 2022-05-26

**Authors:** Jie Du, Ping Zhao, Jiazhen Wang, Sanyuan Ma, Lunguang Yao, Xuwei Zhu, Xinfeng Yang, Xian Zhang, Zhenbo Sun, Shimei Liang, Dongxu Xing, Jianping Duan

**Affiliations:** ^1^ Henan Key Laboratory of Funiu Mountain Insect Biology, College of Life Science and Agricultural Engineering, Nanyang Normal University, Nanyang, China; ^2^ State Key Laboratory of Silkworm Genome Biology, Southwest University, Chongqing, China; ^3^ Laboratory of Tussah Genetics and Breeding, Henan Institute of Sericulture Science, Zhengzhou, China; ^4^ Sericulture and Agri-Food Research Institute, Guangdong Academy of Agricultural Science, Guangzhou, China

**Keywords:** *Antheraea pernyi*, pupal diapause termination, 20-hydroxyecdysone, pupal-adult transition, comprehensive transcriptome

## Abstract

The pupal diapause of univoltine *Antheraea pernyi* hampers sericultural and biotechnological applications, which requires a high eclosion incidence after artificial diapause termination to ensure production of enough eggs. The effect of pupal diapause termination using 20-hydroxyecdysone (20E) on the eclosion incidence has not been well-documented in *A. pernyi*. Here, the dosage of injected 20E was optimized to efficiently terminate pupal diapause of *A. pernyi*, showing that inappropriate dosage of 20E can cause pupal lethality and a low eclosion incidence. The optimal ratio of 20E to 1-month-old pupae was determined as 6 μg/g. Morphological changes showed visible tissue dissociation at 3 days post-injection (dpi) and eye pigmentation at 5 dpi. Comprehensive transcriptome analysis identified 1,355/1,592, 494/203, 584/297, and 1,238/1,404 upregulated and downregulated genes at 1, 3, 6, and 9 dpi, respectively. The 117 genes enriched in the information processing pathways of “signal transduction” and “signaling molecules and interaction” were upregulated at 1 and 3 dpi, including the genes involved in FOXO signaling pathway. One chitinase, three trehalase, and five cathepsin genes related to energy metabolism and tissue dissociation showed high expression levels at the early stage, which were different from the upregulated expression of four other chitinase genes at the later stage. Simultaneously, the expression of several genes involved in molting hormone biosynthesis was also activated between 1 and 3 dpi. qRT-PCR further verified the expression patterns of two ecdysone receptor genes (*EcRB1* and *USP*) and four downstream response genes (*E93*, *Br-C*, *βFTZ-F1*, and *cathepsin L*) at the pupal and pharate stages, respectively. Taken together, these genes serve as a resource for unraveling the mechanism underlying pupal-adult transition; these findings facilitate rearing of larvae more than once a year and biotechnological development through efficient termination of pupal diapause in *A. pernyi* in approximately half a month.

## Introduction

Diapause is, evolutionarily, a physiological state of developmental arrest used by many insects to adapt to environmental changes in their habitats (Denlinger, 2002; Bale and Hayward, 2010). As an adaptive strategy, diapause is common at different developmental stages, such as eggs, larvae, pupae, and adults. Each insect enters diapause at a fixed stage. In *Bombyx mori*, diapause occurs during the embryonic phase; diapause termination of the daughter eggs occurs *via* incubating the developing mother eggs at 15°C, which considerably enhances the establishment of germline transformation technology (Zhao et al., 2012). In *Antheraea pernyi*, diapause occurs during the pupal stage. Previous studies have used 20-hydroxyecdysone (20E) to terminate pupal diapause of *A. pernyi* (Liu Y. et al., 2015; Li et al., 2020). However, the eclosion incidence, a key factor, has never been discussed in these studies, which limits the application of 20E in production and biotechnology development. A high eclosion incidence after pupal diapause termination can easily raise the rearing frequency per year of *A. pernyi*, which can promote the development of sericulture and basic research, including the establishment of germline transformation technology.

Environmental cues usually trigger diapause in insects. Notably, photoperiod and temperature cues (Bale and Hayward, 2010) received by the brain subsequently affect the secretion of molting hormone from the prothoracic glands (PGs) (Mizoguchi et al., 2013). PGs, mediated by several Halloween genes, first produce ecdysone (E), the precursor of the major molting hormone (Gilbert, 2004; Rewitz et al., 2013). E is released into hemolymph, and then transformed into its active form, 20-hydroxyecdysone (20E), as catalyzed by 20-monooxygenase in peripheral tissues (Petryk et al., 2003). The inactivation of PGs and the resulting decline in ecdysteroid titer during the wandering stage causes pupal diapause (Zdarek and Denlinger, 1987; Mizoguchi et al., 2013). Low ecdysteroid titers in the hemolymph of pupae are closely related to pupal diapause maintenance (Smith et al., 1996; Takeda et al., 1997); injection of exogenous 20E into diapausing pupae can terminate pupal diapause (Liu Y. et al., 2015; Reynolds et al., 2019). Many studies have also invoked 20E to reboot pupal-adult transition by artificially increasing 20E content in the hemolymph (Yang et al., 2010; Niitsu et al., 2014; Chen et al., 2016). However, pupal diapause termination *via* the injection of 20E has a dose-dependent effect; injection at a large dose causes mortality in pupae (Williams, 1968; Wu et al., 1994; Chen et al., 2016). This situation limits the efficient use of 20E-induced pupal diapause termination in preparing sufficient number of eggs for production in sericulture and biotechnological development in *A. pernyi*. To date, studies have only determined the effective dosage of 20E to terminate diapause in 18-week-old pupae of *A. pernyi* (Liu Y. et al., 2015). Whether this dosage is also suitable for diapause termination in pupae at other ages and consequently results in a high eclosion incidence remains unclear.

A single ecdysone pulse in the hemolymph can trigger molting and metamorphosis in a stage- and tissue-specific manner (Lan et al., 1999; Rewitz et al., 2013; Yamanaka et al., 2013). However, studies have not clearly documented the genetic expression trend associated with the pupal-adult transition in *A. pernyi* pupae, as triggered by 20E. *E93* is a 20E primary-response gene; its expression can be suppressed by the juvenile hormone (JH) *via* the JH primary-response gene *Kr-h1* during the larval stage (Baehrecke and Thummel, 1995; Urena et al., 2014). However, during pupal-adult metamorphosis, E93 represses JH signaling (Urena et al., 2014; Liu X. et al., 2015). These studies demonstrated that E93 is a universal adult specifier for insects, promoting larval tissue dissociation and adult tissue formation (Mou et al., 2012; Urena et al., 2014; Liu X. et al., 2015). βFTZ-F1 is another 20E downstream nuclear receptor. In *Drosophila*, βFTZ-F1 is involved in fat body dissociation by regulating the expression of matrix metalloproteinase 2 (MMP2) (Bond et al., 2011); the knockdown of *βFTZ-F1* during the late pupal stage suppresses the normal development of adult organs (Sultan et al., 2014). Fat body remodeling is a premise to form normal adults, which involves the disruption of the extracellular matrix in the fat body and causes polygonal fat cells to dissociate into spherical fat cells (Nelliot et al., 2006; Zheng et al., 2016). Cathepsin genes (*Cat*s) in *A. pernyi* (Sun et al., 2017; Sun et al., 2018), *B. mori* (Lee et al., 2009), and *Helicoverpa armigera* (Zhang et al., 2013) also regulate this process. In *Apis mellifera*, pupal-adult transition can be divided into two phases: pupae and pharate adult (Soares et al., 2013). The pharate adult develops underneath the pupal cuticle in the duration between pupal apolysis and adult ecdysis. Eye pigmentation begins at the end of pupal apolysis, and can be used as an indicator for the beginning of pharate adult to assist understanding the pupal-adult transition. In this study, we determined the optimal dosage of 20E and its effect on pupal diapause termination *via* morphological observation and comparative transcriptome analysis to improve the understanding of possible biological processes during pupal-adult transition in *A. pernyi*.

## Materials and Methods

### Experimental Insect

The larvae of univoltine *A. pernyi* were fed on oak trees in the field. In July 2020, diapause pupae at the age of 1 month were directly used for a pretest, and the sex was not determined. A replicate experiment was conducted in 2021. To confirm the diapause stage as being the same for the experimental pupae, the larvae, which started spinning at the same time, were chosen for preparing prepupae. After the prepupae were formed in early June, the cocoons were immediately transferred to the laboratory and held at 25 ± 1°C with a photoperiod of light: dark (LD) 12:12 until used.

### Pupal Diapause Termination Triggered by 20E

Commercially available 20E from Sangon Biotech (Shanghai, China) was used to terminate pupal diapause. To optimize the injection dosage, a weighed amount of 20E was first dissolved in 1 part absolute alcohol and then diluted with 99 parts pure water (1%) to prepare a 4 μg/μL solution, which was injected using a finely drawn glass capillary. The injection dosage was converted to mass ratios of 4 μg 20E per Gram of pupa (4 μg/g), 6 μg/g, and 8 μg/g to treat the diapause pupae. Controls were treated with phosphate-buffered saline. After treatment, the injected pupae were stored in an incubator at 25 ± 1°C and 85% ± 5% relative humidity with a photoperiod of LD 12:12. To assess the effects of diapause termination, the incidences of diapause termination, eclosion, and mating were counted. The dosage corresponding to the highest eclosion and mating incidences was considered optimal. Each experiment consisted of three replicates with 10 individuals per replicate. During this process, the pupal cuticle was dissected and removed as carefully as possible in normal saline, and the tissue development process underneath the pupal cuticle was recorded after removing the cuticle under a stereoscope (Nikon, Japan).

### Sample Collection, RNA Isolation, and Transcriptome Sequencing

To further explore the global dynamics of gene expression during pupal-adult transition, the one-month-old pupae were induced by the above optimal dosage of 20E to prepare the samples for transcriptome sequencing. The pupae were collected at 0, 1, 3, 6, and 9 days post-injection. Three pupae were mixed in a single sample after the removal of pupal cuticle. Three samples were prepared at each time point as biological replicates. Total RNA was extracted from 15 samples using TRIzol reagent (Invitrogen, United States) following the manufacturer’s protocol. RNA purity and integrity were confirmed using a NanoPhotometer^®^ spectrophotometer (IMPLEN, United States) and an Agilent Bioanalyzer 2100 system (Agilent, United States), respectively. A total of 3 μg of RNA from each sample was used to construct a sequencing library. Fifteen sequencing libraries were generated using a VAHTS mRNA-seq V2 Library Prep Kit (Vazyme, China) following the manufacturer’s recommendations and details (Lei et al., 2021). Paired-end sequencing was carried out on an Illumina HiSeq™ 2500 platform (Illumina, United States) at Suzhou Transcriptome Biotechnology Co., Ltd. (Suzhou, China).

### Identification of Differential Expressed Genes

Clean reads were generated by removing adapters and low-quality reads from raw data using Trimmomatic v0.36 (Bolger et al., 2014). All clean reads were aligned to the reference genome of *A. pernyi* (Duan et al., 2020) using HISAT2 v2.2.1 (Zhang et al., 2021). DEGs were identified by pairwise comparisons at adjacent time points (1 dpi versus 0 dpi, 3 dpi versus 1 dpi, 6 dpi versus 3 dpi, and 9 dpi versus 6 dpi) using the DESeq2 package in R (Love et al., 2014). The significance of the differential gene expression was assessed based on the following thresholds after 20E treatment: absolute value of log2-fold change (|Log2FC|) ≥ 1 and false discovery rate (FDR) < 0.05.

### Functional Enrichment Analysis of DEGs

To assess the biological function and signaling pathways involved in the differential gene expression trend, the DEGs at each time point, as compared to their former time points, were subjected to enrichment analysis with the GO (http://www.geneontology.org/) and KEGG (https://www.kegg.jp/) databases using the ClusterProfile package (Wu et al., 2021). Adjusted *p*-values < 0.05 and Q-values < 0.05 were set as the cutoff criteria.

### Time-Series Analysis of Gene Expression

The STEM clustering method (Ernst and Bar-Joseph, 2006) was used to construct gene expression profiles using data from the time-course samples after treatment. During the process, a permutation test was applied to determine the significance of the profiles. The profiles with an FDR <0.05 were significantly clustered. Functional enrichment analysis was performed to understand the biological functions of the significantly clustered profiles, as described above.

### Quantitative Real-Time PCR

First-strand cDNAs of the samples were synthesized at each time point using the BeyoRT II cDNA Synthesis Kit with the gDNA Eraser (Beyotime, China). qRT-PCR was performed with an SYBR Green qPCR Mix (Toyobo, Japan) using an ABI 7500 Fast Real-Time PCR System (Applied Biosystems, United States) to detect the relative expression levels of the genes related to adult development. The qRT-PCR conditions were described in Lei et al. (Lei et al., 2021). Each assay was performed in triplicate. The glyceraldehyde 3-phosphate dehydrogenase gene (*GAPDH*) in *A. pernyi* was used as the control (Lei et al., 2021). Supplementary Table S1 lists all of the primers used for qRT-PCR.

### Statistical Analysis

Data analysis was performed using GraphPad Prism 5.0.1 (GraphPad, United States). The cumulative eclosion incidence was fitted using the nonlinear function curve. The qRT-PCR results and the incidences of diapause termination, eclosion, and mating after 20E treatment were compared using one-way Analysis of Variance (ANOVA). *p* < 0.05 was set as the criterion of statistical significance.

## Results

### Determination of Optimal 20E Dosage for Pupal Diapause Termination

**FIGURE 1:**
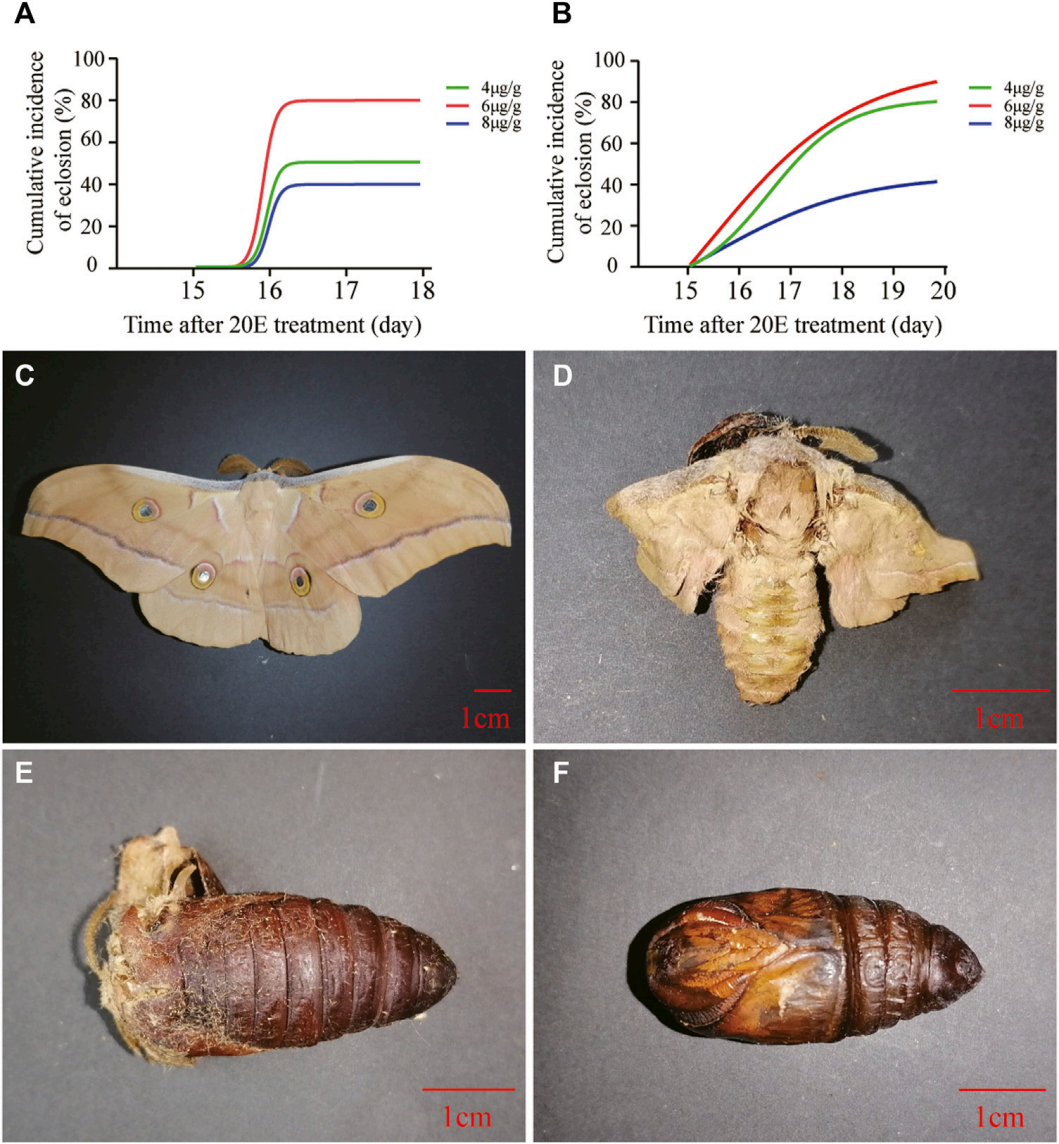
Effect of 20E on the development of diapause pupae at the age of 1 month. **(A)** Cumulative eclosion incidence of *A. pernyi* pupae after treatment using different dosages of 20E in July 2020. **(B)** Cumulative eclosion incidence of *A. pernyi* pernyi pupae after treatment using different dosages of 20E in July 2021. Colored lines represent different treatments. **(C)** A normal moth induced by 20E. **(D)** An abnormal moth induced by 20E with unsuccessful copulation. **(E)** Pupal-adult death induced by 20E. **(F)** Pupal death induced by 20E.

Two experiments were performed to determine the optimal dosage of 20E for pupal diapause termination at the age of 1 month. The pretest in 2020 showed that the ratio of 20E to pupal weight at a dose of 6 μg/g triggered the pupal-adult transition with the highest eclosion incidence, which obviously decreased at a high dose of 8 μg/g (Figure 1A). Based on the pretest results, a replicate experiment was carried out in the following year. The results showed that the cumulative eclosion incidence of 6 μg/g was the highest, whereas 8 μg/g caused the lowest cumulative eclosion incidence (Figure 1B). Additionally, phenotypic observations showed that abnormal development was induced at different dosages. The abnormalities ranged from slightly abnormal moths (Figure 1D) to extremely high pupal death (Figure 1F), including pupal-adult death (Figure 1E). The 8-μg/g dose caused relatively severe abnormalities.

**FIGURE 2:**
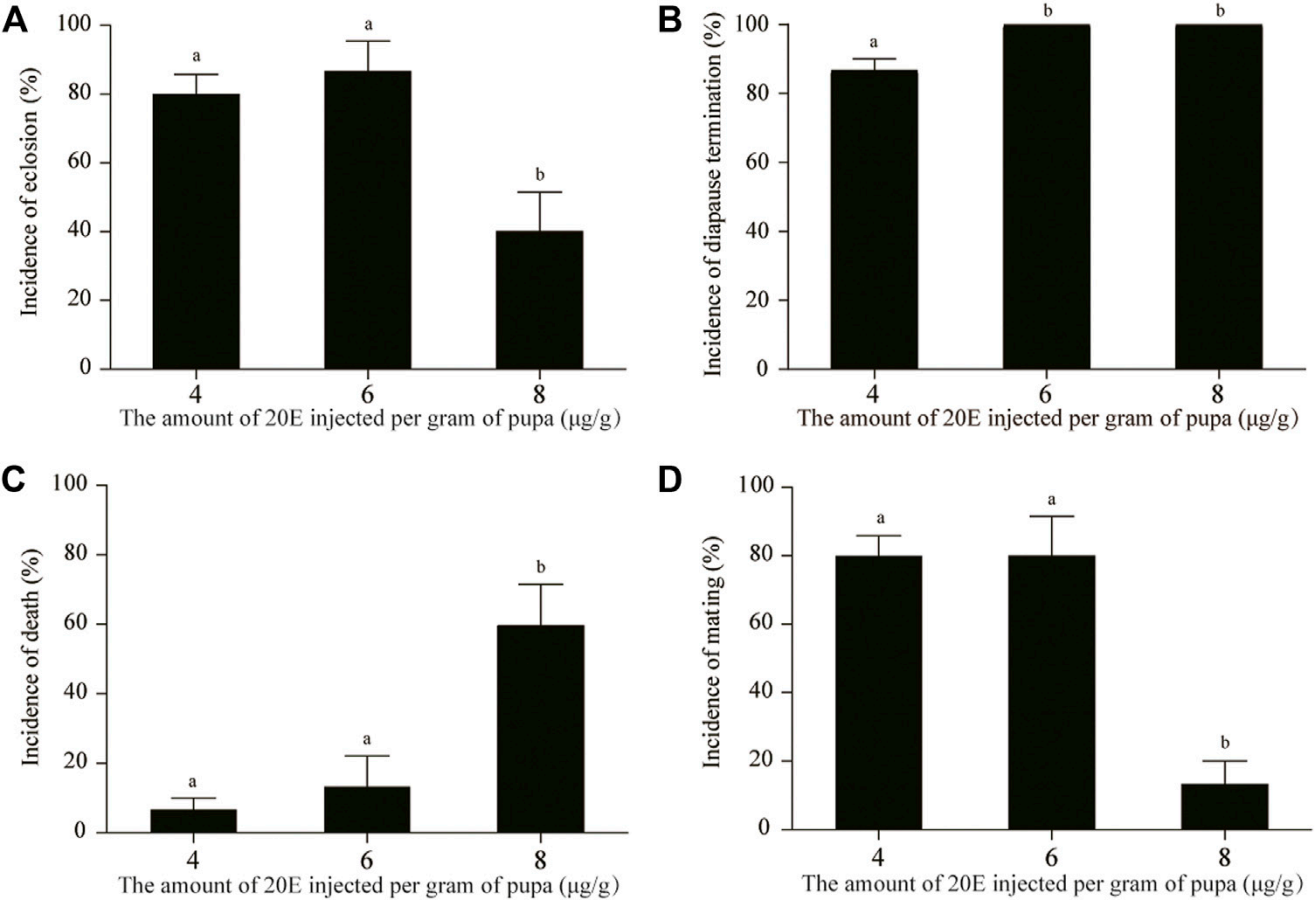
Comparative analysis of the incidences of eclosion **(A)**, diapause termination **(B)**, death **(C)**, and mating **(D)** after treatment using different doses of 20E. Death included pupal death and pupal-adult death. Different lowercase letters indicate significant differences (*p* < 0.05, ANOVA); identical lowercase letters show that there is no significant difference (*p* > 0.05, ANOVA).

Statistical analysis showed that the eclosion incidences at 4 and 6 μg/g 20E were not significantly different (Figure 2A), but their diapause termination incidences were significantly different (Figure 2B). The diapause state of all pupae at the age of 1 month could be broken at the dosage of 6 μg/g. Although the diapause termination incidence of 8 μg/g also reached 100% (Figure 2B), the death incidence was very high (Figure 2C) and the eclosion and mating incidences were significantly reduced (Figure 2A,D). Approximately half of the moths induced by 8 μg/g did not copulate.

### Progressive Tissue Development Process Induced by 6 μg/g 20E

**FIGURE 3:**
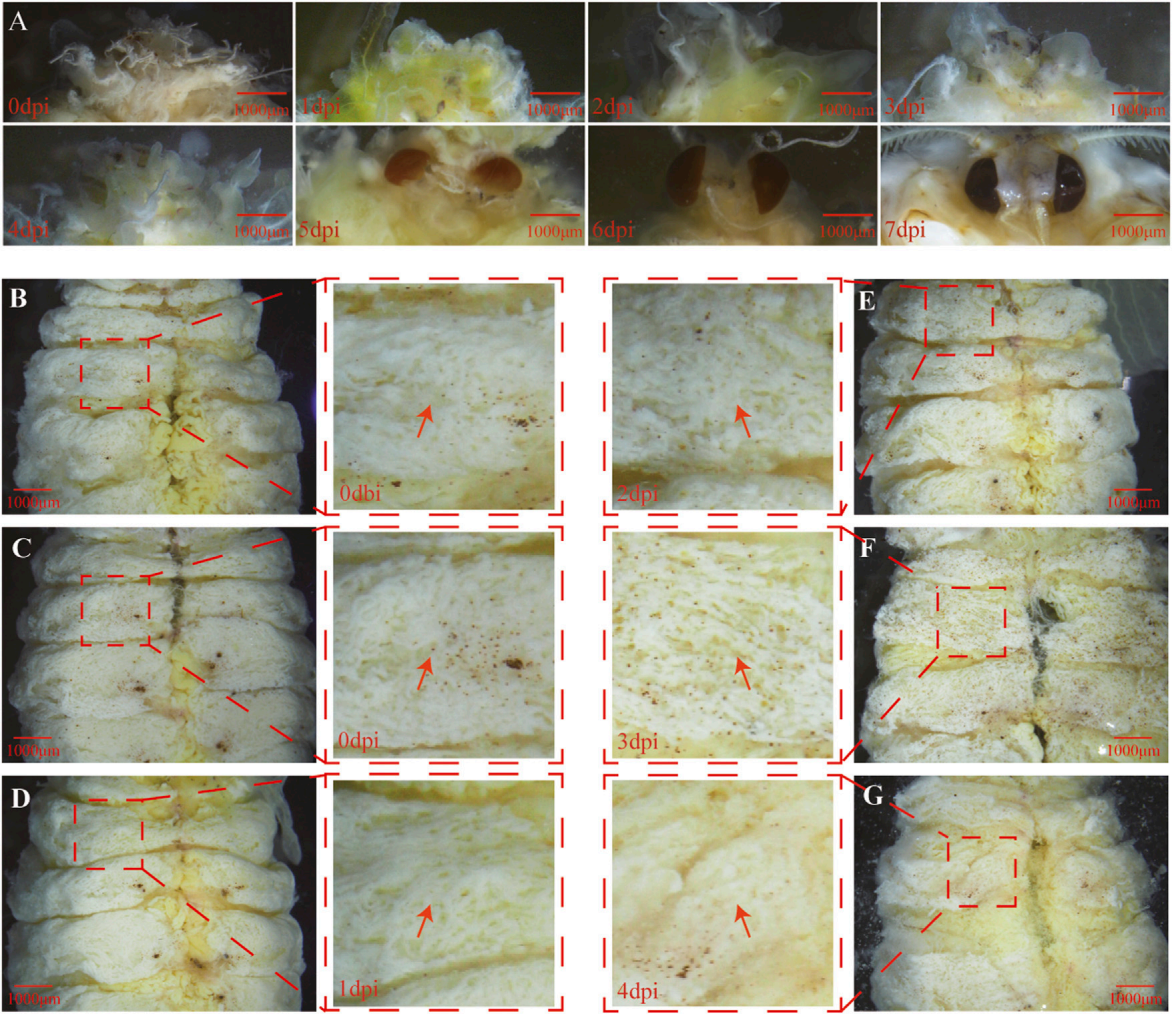
Progressive tissue development process triggered by 6 μg/g 20E. The eye pigmentation process from 0 to 7 dpi was documented **(A)**. After carefully removing the pupal cuticle, the abdominal tissue underneath the cuticle at 0 dbi **(B)**, 0 dpi **(C)**, 1 dpi **(D)**, 2 dpi **(E)**, 3 dpi **(F)**, and 4 dpi **(G)** was directly observed. Partially magnified images of the abdominal segments show that visible fat body dissociation emerged at 3 dpi, which is shown using a red arrow. The dpi and dbi represent the acronyms of day post and before injection, respectively.

To understand the transition between the pupae and pharate adult, the developmental process of tissue underneath the pupal cuticle was documented immediately after removing the cuticle. The time-course observation showed that eye pigmentation appeared at 5 dpi (Figure 3A), indicating that *A. pernyi* was in the pupal state between 0 dpi and 4 dpi when the larval fat body might be sharply dissociating, after which *A. pernyi* entered the pharate-adult stage. Tissue dissociation was clearly visible at 3 dpi (Figure 3F) and more evident at 4 dpi (Figure 3G).

### General Gene Expression After Pupal Diapause Termination

**FIGURE 4:**
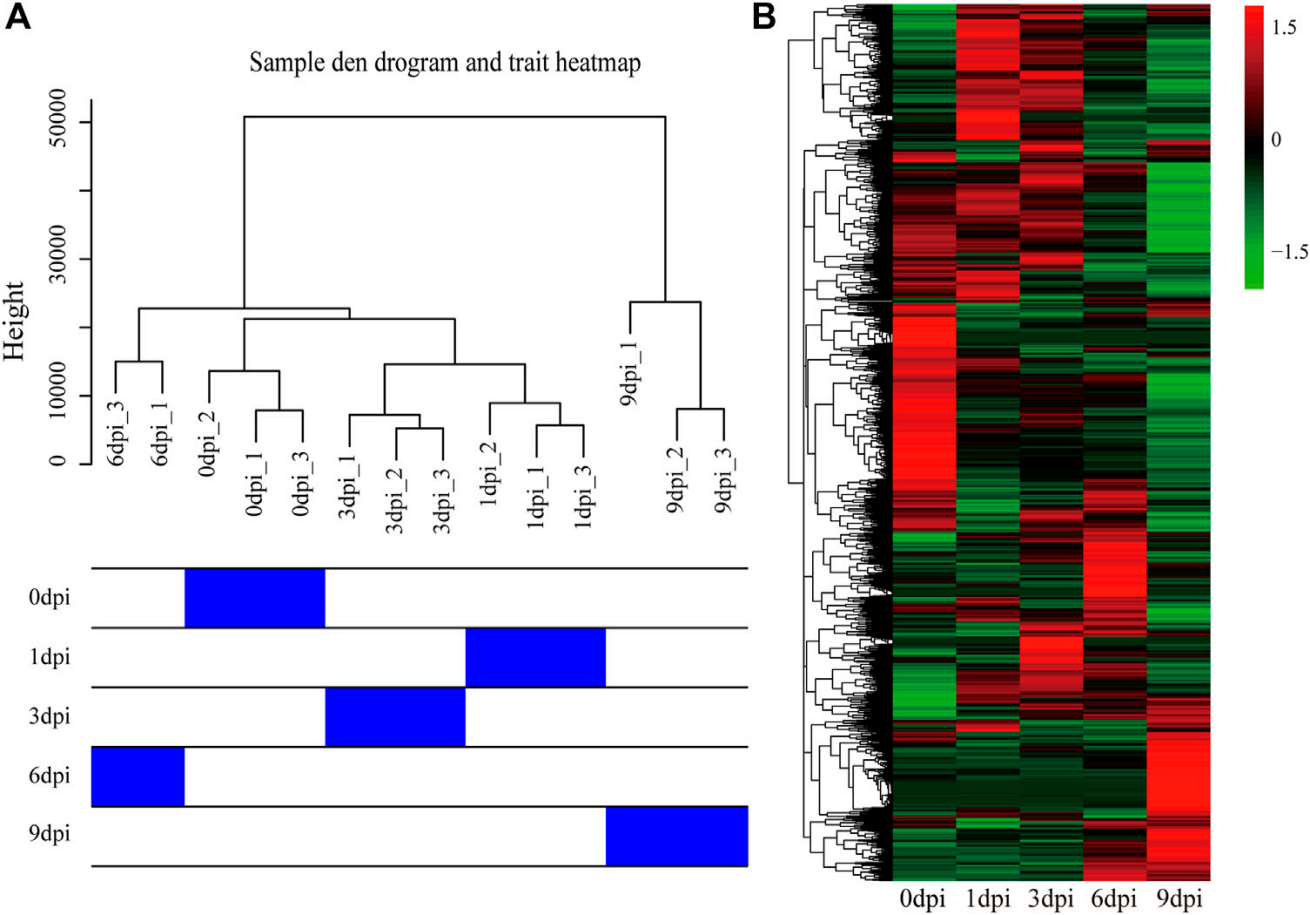
Hierarchical cluster of the biological replicates and all genes using transcriptome data. **(A)** Relationship between the replicates from the five stages of 20E-treated pupae in *A. pernyi*. **(B)** Results of the hierarchical cluster analysis for all obtained genes.

Transcriptome analysis of the time-course samples after pupal diapause termination can provide insights into the molecular mechanisms underlying pupal-adult transition. To explore the global dynamics of gene expression, we performed transcriptome sequencing using total RNA isolated from the five stages of the pupal-adult transition. Each stage contained three independent biological replicates (15 samples in total). A total of 0.87 billion high-quality reads (average of ∼58 million reads from each sample) were generated for the 20E-treated pupae (Supplementary Table S2). The read counts were first transformed into FPKMs and then used for hierarchical cluster analysis based on the Euclidean distance. The clustering results suggested an abnormal replicate at the 6 dpi. After removing the abnormal replicate (6dpi_2), the hierarchical cluster of the remaining samples showed high-quality biological replicates at the same developmental stages (Figure 4A). Differences in the gene expression between the stages were visualized based on the FPKM values of 18,862 genes (Figure 4B).

### Differential Gene Expression Dynamics After Pupal Diapause Termination

**FIGURE 5:**
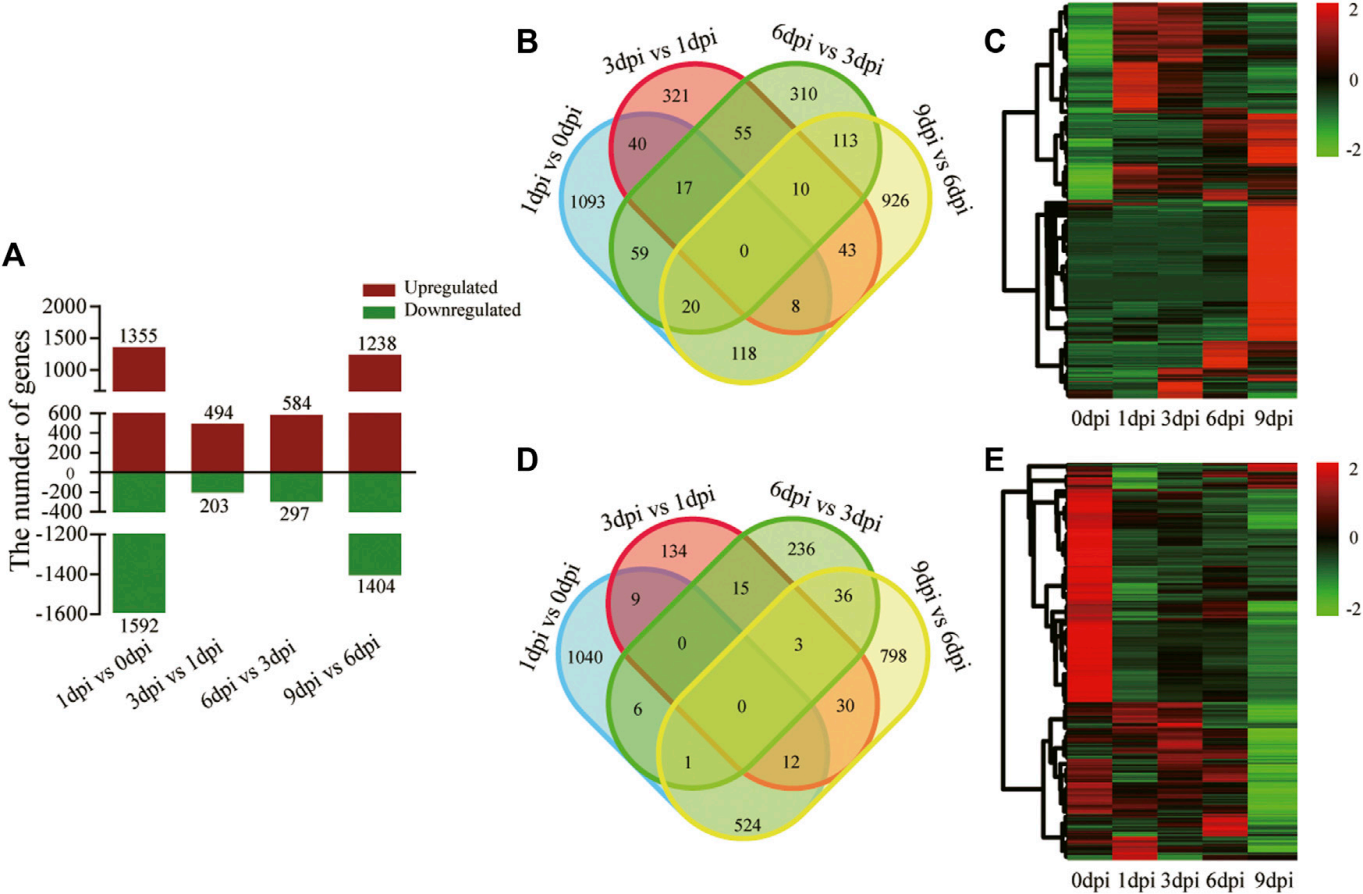
Differentially expressed genes in four pairwise sampling stages after 20E treatment. **(A)** Number of upregulated and downregulated genes in the four pairwise stages of 1 dpi versus 0 dpi, 3 dpi versus 1 dpi, 6 dpi versus 3 dpi, and 9 dpi versus 6 dpi. **(B)** Venn diagram of the upregulated genes in the four pairwise stages. **(C)** Heatmap showing the expression profile of the upregulated genes in the time-course developmental stages. **(D)** Venn diagram of the downregulated genes in the four pairwise stages. **(E)** Heatmap showing the expression profile of the downregulated genes in the time-course developmental stages.

Differentially expressed genes (DEGs) were identified using pairwise sample comparisons between two adjacent time points. Nearly one-third of the genes (5,235 out of 18,862) were differentially expressed at a minimum of one time point (Figure 5A). Relative to 0 dpi, 1,355 upregulated and 1,592 downregulated genes were identified at 1 dpi (Supplementary Table S3). Compared with 1 dpi, there were 494 upregulated and 203 downregulated genes (494/203 DEGs) at 3 dpi, respectively (Supplementary Table S4). Additionally, 584/297 and 1,238/1,404 DEGs were identified in the comparisons of 6 dpi versus 3 dpi (Supplementary Table S5) and 9 dpi versus 6 dpi (Supplementary Table S6), respectively (Figure 5A). The most significant change in the gene expression profile was observed in the comparisons of 1 dpi versus 0 dpi (2,947 in total) and 9 dpi versus 6 dpi (2,642 in total). The fewest DEGs (697 in total) were found at 3 dpi compared with 1 dpi. These results showed a smaller number of DEGs middle in pupal-adult transition than that early and later in pupal-adult transition, further indicating that early events triggering the transition happened before 5 dpi when *A. pernyi* started the pharate state.

Furthermore, we separately depicted the upregulated (Figure 5B,C) and downregulated (Figure 5D,E) DEGs. The number of stage-preferential upregulated and downregulated genes clearly varied. There were 1,093/1,040, 321/134, 310/236, and 926/798 stage-preferential DEGs identified in the four sampling stages after 20E treatment. These genes suggested some independent developmental programs for each stage during the pupal-adult transition.

### Functional Enrichment Analysis of DEGs in Each Pairwise Comparison

To further mirror the critical interval of the pupal-adult transition at the molecular level, the Gene Ontology (GO) and Kyoto Encyclopedia of Genes and Genomes (KEGG) enrichment analyses were performed. Almost all of the DEGs in 3 dpi versus 1 dpi and 6 dpi versus 3 dpi were annotated to have no relationship with the terms of transcription, translation, replication, and repair; only 1 DEG in 6 dpi versus 3 dpi was annotated in the terms of “transcription” (Supplementary Figure S1). The GO enrichment analysis also showed a significant difference in the top five terms of the cellular component category. In 3 dpi versus 1 dpi and 6 dpi versus 3 dpi, “extracellular region,” “intrinsic component of membrane,” “membrane part,” “integral component of membrane,” and “membrane” were significantly enriched, which were different from the top terms underlying 1 dpi versus 0 dpi and 9 dpi versus 6 dpi (Supplementary Figure S2). These differences in the molecular functions suggested that some sharp tissue remodeling occurred in the interval between 1 and 6 dpi.

### Time-Series Analysis of Gene Expression

**FIGURE 6:**
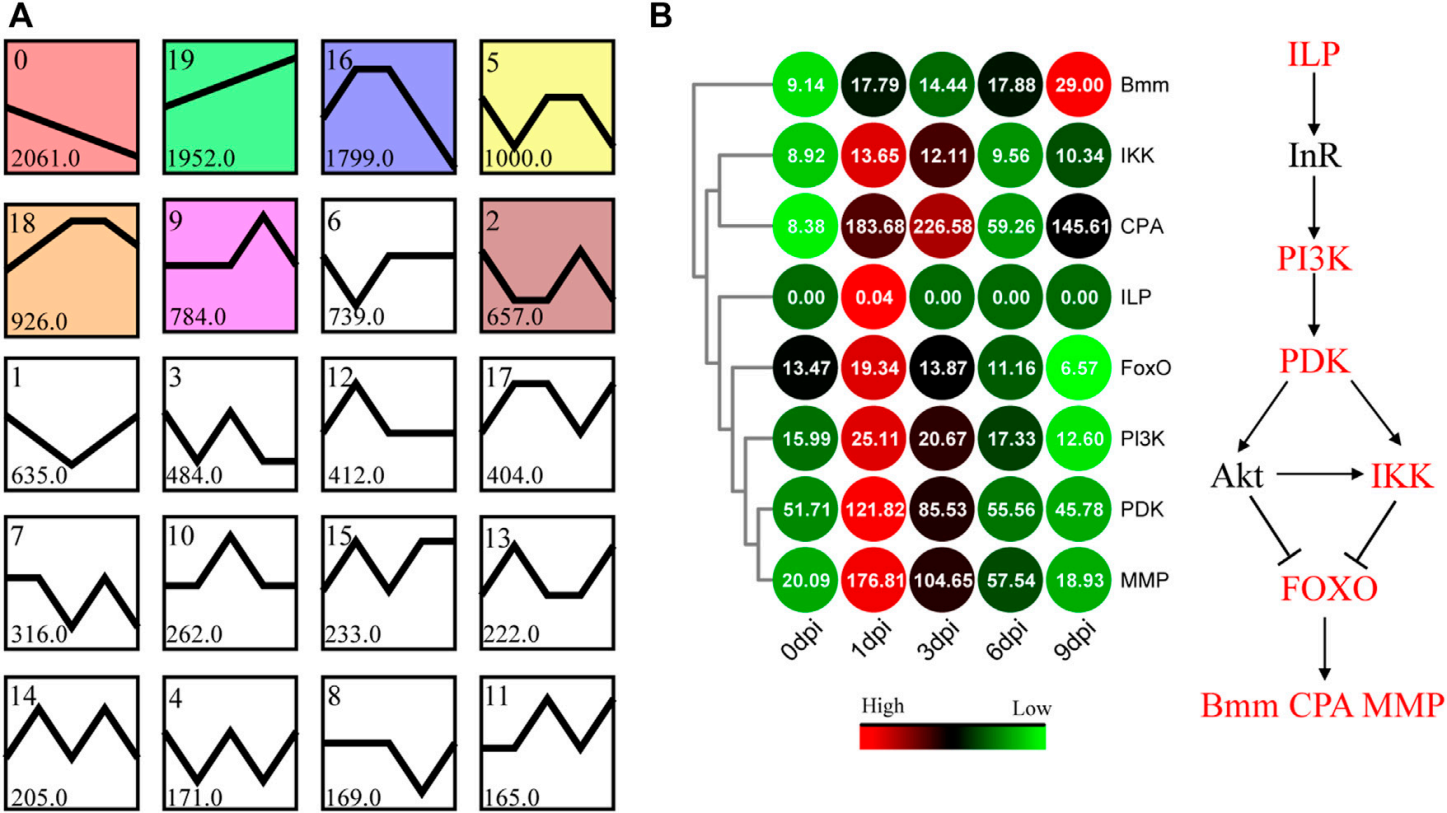
Patterns of gene expression across different time points inferred by the STEM and enrichment analyses. **(A)** Twenty candidate profiles obtained by the STEM analysis. The six colored profiles are significant profiles (FDR <0.05). **(B)** Expression pattern of the genes involved in the FOXO signaling pathway. Bmm, CPA, and MMP represent the glyceride lipase Brummer, molting fluid carboxypeptidase A, and matrix metalloproteinase, respectively.

To identify the major transcriptional dynamics associated with tissue dissociation during the pupal-adult transition, a short time-series expression miner (STEM) analysis was applied to group genes with similar expression profiles. Twenty candidate profiles were obtained, seven of which were significant (FDR <0.05; Figure 6A).

**FIGURE 7:**
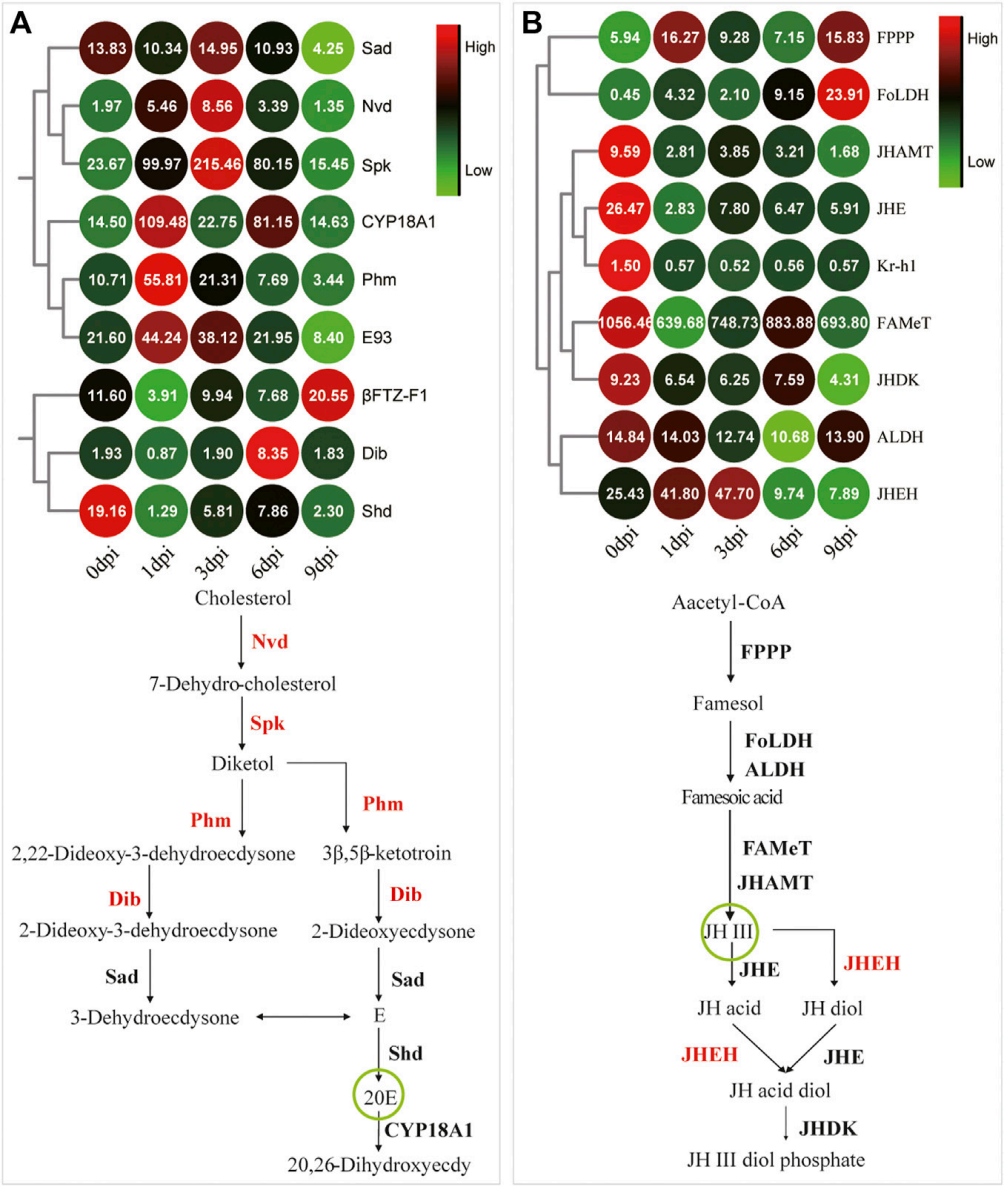
Coordinated expression changes in the genes related to insect hormone metabolism. **(A)** The expression pattern of the Halloween genes that mediate molting hormone biosynthesis. *βFTZ-F1* and *E93* are two downstream response genes of the molting hormone signaling pathway. CYP18A1 is the key enzyme of 20E inactivation. The remaining genes are Halloween genes for molting hormone metabolism. The green circle shows the active form of ecdysone. **(B)** The gene expression pattern involved in JH metabolism. *Kr-h1* is a downstream response gene of the JH signaling pathway. The green circle shows the active form of JH.

Based on the visible tissue dissociation at 3 dpi (Figure 3), we hypothesized that the key change in the genetic regulation signals underlying the dissociation should have occurred before 3 dpi. Immediately thereafter, KEGG enrichment analysis was performed using the genes in Profile 16. A total of 117 genes were annotated, i.e., involvement in “signal transduction” and “signaling molecules and interaction” (Supplementary Figure S3). Especially, the expression of genes involved in the FOXO signaling pathway (Figure 6B, Supplementary Table S7) and molting hormone biosynthesis (Figure 7, Supplementary Table S8) was upregulated. In addition, we also found that several important genes, including the genes of triglyceride lipase Brummer (*Bmm*), molting fluid carboxypeptidase A (*CPA*), matix metalloproteinase (*MMP*) and two 20E downstream transcriptional factors, *E93* and *βFTZ-F1*, were all induced at different stages.

### Expression Validation of Important Genes Related to Pupal-Adult Transition

**FIGURE 8:**
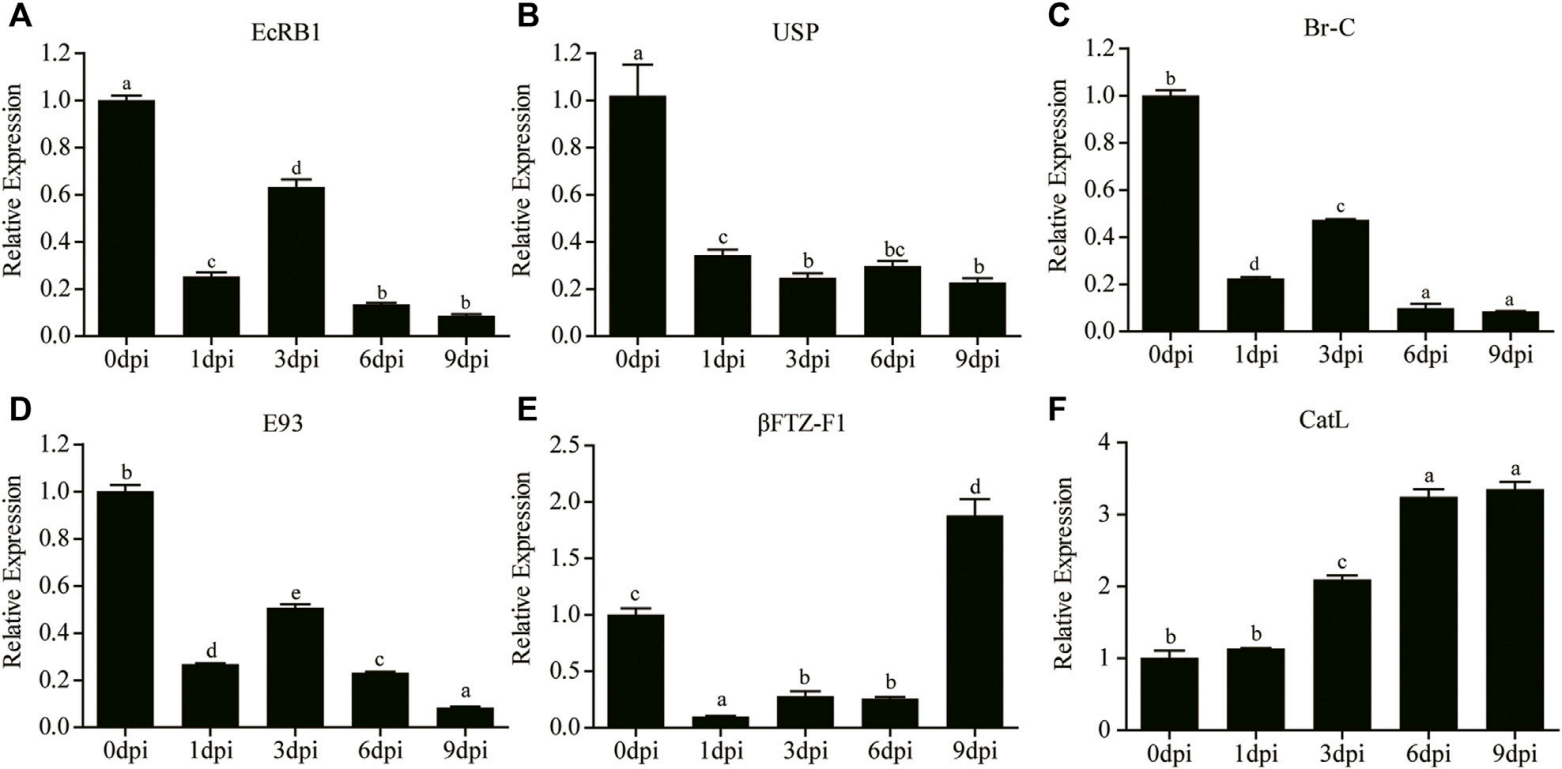
Relative expression levels of *EcRB1* **(A)**, *USP* **(B)**, *Br-C* **(C)**, *E93* **(D)**, *βFTZ-F1* **(E)**, and *CatL* **(F)** at different time points were determined by qRT-PCR. Different lowercase letters indicate significant difference (*p* < 0.05, ANOVA).

Two upstream receptor genes and four downstream response genes of the ecdysone signaling pathway were selected for qRT-PCR analysis. The results showed that the expression of ecdysone receptor B1 gene (*EcRB1*) was downregulated at 1 dpi and increased at 3 dpi, which was different from the continuously low expression of the ultraspiracle gene (*USP*) after 20E treatment (Figure 8A,B). The expression patterns of two early response genes, *E93* and *Br-C*, were consistent with the trend of *EcRB1* (Figure 8C,D). In addition, the expression of *βFTZ-F1* was significantly downregulated at 1 dpi, and then significantly increased to a high level at 9 dpi (Figure 8E). Significantly upregulated expression of *CatL* was also observed after 3 dpi (Figure 8F).

## Discussion

The univoltine *A. pernyi* has a long pupal diapause period. Efficient pupal diapause termination would improve the development of production and biotechnology. Earlier studies demonstrated that 20E treatment or photoperiod manipulation can terminate pupal diapause in *A. pernyi* (Takeda et al., 1997; Liu Y. et al., 2015). The photoperiodic treatment lasts about 20 days, after which the treated pupae enter the post-termination phase (Li et al., 2020). Our results showed that 20E treatment efficiently led to adult ecdysis approximately half a month later (Figure 1). Therefore, 20E treatment remains convenient for pupal diapause termination at different diapause stages. A high dose of 20E can result in death of pupae and emergence failure (Williams, 1968; Wu et al., 1994), which occurs in *A. pernyi*. Here, we first documented the malformation effect triggered by a high dose of 20E in *A. pernyi* (Figure 2). Inappropriate dosage can result in slightly or extremely abnormal development. For the purpose of a high eclosion incidence, we determined the optimal dose of 20E (6 μg/g) for pupal diapause termination at the age of 1 month (Figures 1, 2). Based on another optimal dose of 20E (2 μg/g) for the diapause termination in 4-month-old pupae (Supplementary Figure S4), comprehensive consideration showed that how much 20E was needed to efficiently break pupal diapause depended on when 20E was administered during diapause. A number of different stages should be further included to show a dose response curve during diapause, which could optimize the 20E application in the future. Different natural environments affect the pupal diapause intensity in *Antheraea* insects (Takeda et al., 1997). The slight difference in the curve fitting of the cumulative eclosion incidences between the two replicates (Figure 1A,B) also confirmed this effect, suggesting slightly stronger diapause tendency of pupae in 2021 than that in 2020.

*Antheraea pernyi* has been extensively used as a classic organism for examining diapause regulation (Williams and Adkisson, 1964; Li et al., 2020). Subsequent studies have indicated that Cathepsins L and O participate in the disruption of the extracellular matrix and regulate fat body dissociation in *A. pernyi* (Sun et al., 2017; Sun et al., 2018). Our results provided more evidence that three other Cathepsins were also induced in a similar manner between 1 and 3 dpi; they likely modulated the dissociation process together (Supplementary Figure S5, Supplementary Table S9). Time-series transcriptional responses could provide biological information of stage-specifically molecular events. The expression of genes in Profile 0 and 19 was continuously downregulated and upregulated, respectively, which might be related to diapause maintenance or development reboot. However, Profile 2, 5, 9, 16, and 18 showed a distinct expression fluctuation, indicating stage-specific events of pupal-adult transition. Further enrichment analysis revealed the involvement of upregulated DEGs in the FOXO signaling pathway in Profile 16 (Figure 6). 20E-induced nuclear localization of FOXO controls lipolysis and fat body dissociation by upregulating the expression of lipase, carboxypeptidase, and metalloproteinase genes (Hossain et al., 2013; Cai et al., 2016; Jia et al., 2017). The upregulated expression of Forkhead box O (*FoxO*), triglyceride lipase Brummer (*Bmm*), molting fluid carboxypeptidase A (*CPA*), and matix metalloproteinase genes (*MMP*) supported the same conclusion for *A. pernyi* (Figure 6). The transcription activity of FOXO depends on its dephosphorylation (Pan et al., 2018), indicating that the upregulated expression of inhibitor of nuclear factor kappa B kinase (IKK) and phosphoinositide-dependent kinase (PDK) fails to induce the cytoplasmic localization of FOXO by phosphorylation early after 20E treatment. 20E might repress the phosphorylation of PDK and IKK at the post-translation level to maintain FOXO nuclear localization, which controls lipolysis and fat body dissociation of *A. pernyi*. Further in-depth studies are warranted.

Many studies have showed that E is first synthesized in PG by ecdysteriodogenic enzymes encoded by several Halloween genes, followed by conversion into 20E in peripheral tissues (Petryk et al., 2003; Gilbert, 2004; Rewitz et al., 2013). Earlier reports documented that 20E pulse is necessary for pupal-adult transformation in *Tenebrio molitor* and *Apis mellifera* (Quennedey et al., 1983; Soares et al., 2013), and a long-day photoperiodic manipulation could trigger two 20E pulses sequentially in the next 35 days in *A. pernyi* pupae (Li et al., 2020). In the present study, the intermittent expression of the 20E inactivation enzyme and the upregulated expression of four Halloween genes (Figure 7) suggested a similar role as that of 20E pulse. We speculated that exogenous 20E indirectly triggered the expression of Halloween genes by a downstream activator factor, followed by the synthesis of endogenous 20E. PG involution is a foregone destination before adult ecdysis (Quennedey et al., 1983; Dai and Gilbert, 1997). Now that Halloween genes can be indirectly induced at 1 and 3 dpi, the degeneration of *A. pernyi* PG may commence later (Figure 3A). Further studies should investigate this degeneration process. Eye pigmentation is an important feature that indicates the beginning of the pharate stage (Shimono et al., 2009; Soares et al., 2013). Between 5 dpi and adult ecdysis, we envisioned that *A. pernyi* is in a pharate state, before which *A. pernyi* sustains the pupal state. The high expression of *βFTZ-F1* during the late pupal stage modulated the normal development of adult organs in *Drosophila* (Sultan et al., 2014). We also observed the high expression of *βFTZ-F1* at 9 dpi (Figure 7A, 8E), indicating the conserved function of *βFTZ-F1* between *A. pernyi* and *Drosophila*. In *B. mori*, FOXO regulates JH degradation by directly binding to the promoter regions of the genes involved in JH degradation (Zeng et al., 2017). In the present study, the expression of the juvenile hormone epoxidase hydrolase gene (*JHEH*) was also upregulated together with *FoxO* (Figure 6B, 7B), suggesting their regulatory relationship. EcRB1 and USP form a heterodimeric nuclear hormone receptor to mediate ecdysteroid action in insects. During pupal development, high 20E titers could repress the expression of *EcRB1* in *Apis mellifera* (Mello et al., 2014). The downregulated expression of *EcRB1* and *USP* at 1 dpi showed the similar repressive effect early after 20E treatment (Figure 8A,B). Similar results had also been reported in the study of 20-day-old pupae in *A. pernyi* (Ru et al., 2017). As two key transcription factors in ecdysone signaling during metamorphosis, the expression of *E93* and *Br-C* are induced by the ternary complex of 20E-EcRB1-USP (Liu X. et al., 2015; Qian et al., 2017). The same expression patterns of *EcRB1*, *E93*, and *BR-C* showed the similar regulation of 20E-EcRB1-USP on *E93* and *Br-C* in *A. pernyi* (Figure 8).

Three trehalase genes were differentially expressed in 1 dpi versus 0 dpi (Supplementary Figure S6, Supplementary Table S10). In some insects, the expression of trehalase genes is modulated by 20E signaling (Yao et al., 2010; Li et al., 2020). Trehalose is an important form of energy during diapause and is also a basic substance for chitin biosynthesis. Acting as a key enzyme of trehalose metabolism, the three trehalases should function within energy metabolism during the early stages of the pupal-adult transition. Chitin is a structural molecule in the insect midgut and exoskeleton. Chitinases located in the midgut and cuticle regulate pathogen invasion and the cyclical renewal of chitin fibers (Noh et al., 2018; Liu et al., 2020). During metamorphosis, chitinase genes act as late response genes of 20E signaling to regulate wing development and the nymph-adult transition (Xu et al., 2020; Wu et al., 2022). The five chitinase genes upregulated at 1, 6, or 9 dpi (Supplementary Figure S6, Supplementary Table S10) may be necessary to promote proper wing development, liquefaction of the old cuticle, and midgut degeneration during adult development. Confirmation of these hypotheses requires further functional analyses.

In summary, the optimal dosage of 20E for diapause termination in the age of 1 month was first determined for a high eclosion incidence. After 20E treatment, genes related to FOXO signaling pathway, molting hormone biosynthesis, energy metabolism, and tissue remodeling were successively reactivated, which sped up pupal-adult metamorphosis in approximately half a month. This study provides a foundation for further mechanistic research on pupal-adult metamorphosis, the rearing of larvae more than once a year, and the establishment of germline transformation technology in *A. pernyi*.

## Data Availability

The datasets presented in this study can be found in online repositories. The names of the repository/repositories and accession number(s) can be found below: https://ngdc.cncb.ac.cn/gsa/, CRA006017.
