# Supplementary figures and images for: Pupal Diapause Termination and Transcriptional Response of Antheraea pernyi (Lepidoptera: Saturniidae) Triggered by 20-Hydroxyecdysone
Source: Front Physiol. 2022 May 26;13:888643. doi: 10.3389/fphys.2022.888643 (PMC9204484; doi:10.3389/fphys.2022.888643)

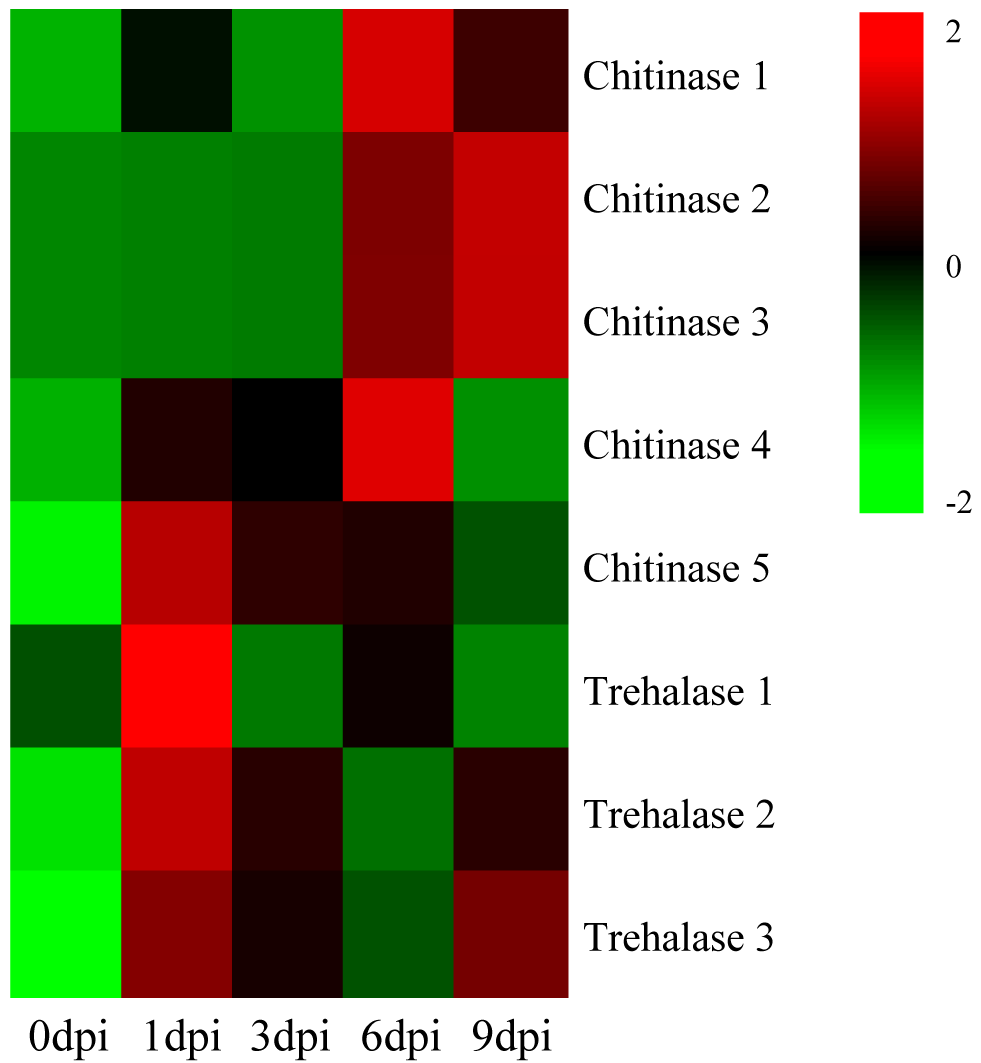

Supplement: Supplementary file 3 [file Image6.TIF]

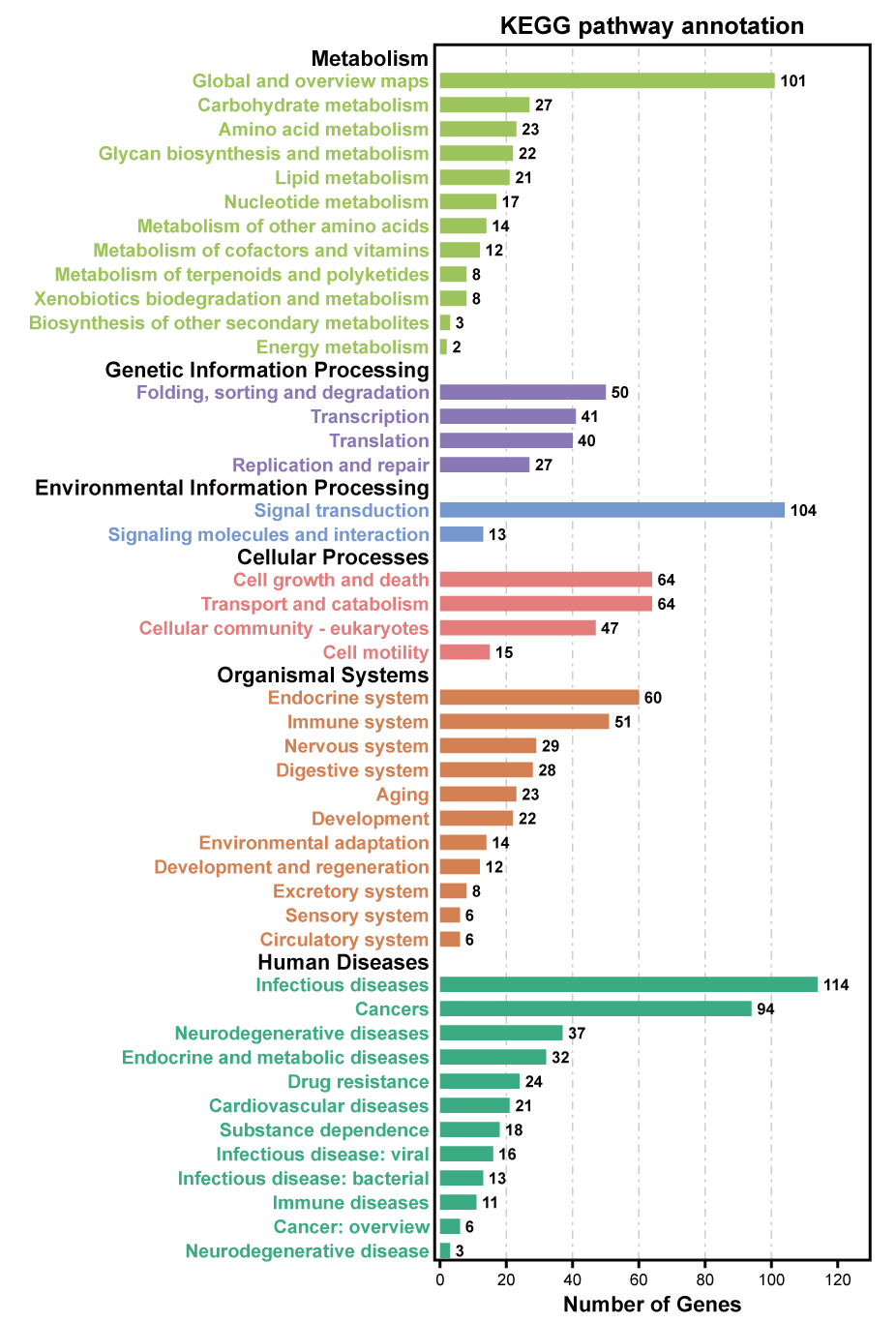

Supplement: Supplementary file 5 [file Image3.TIF]

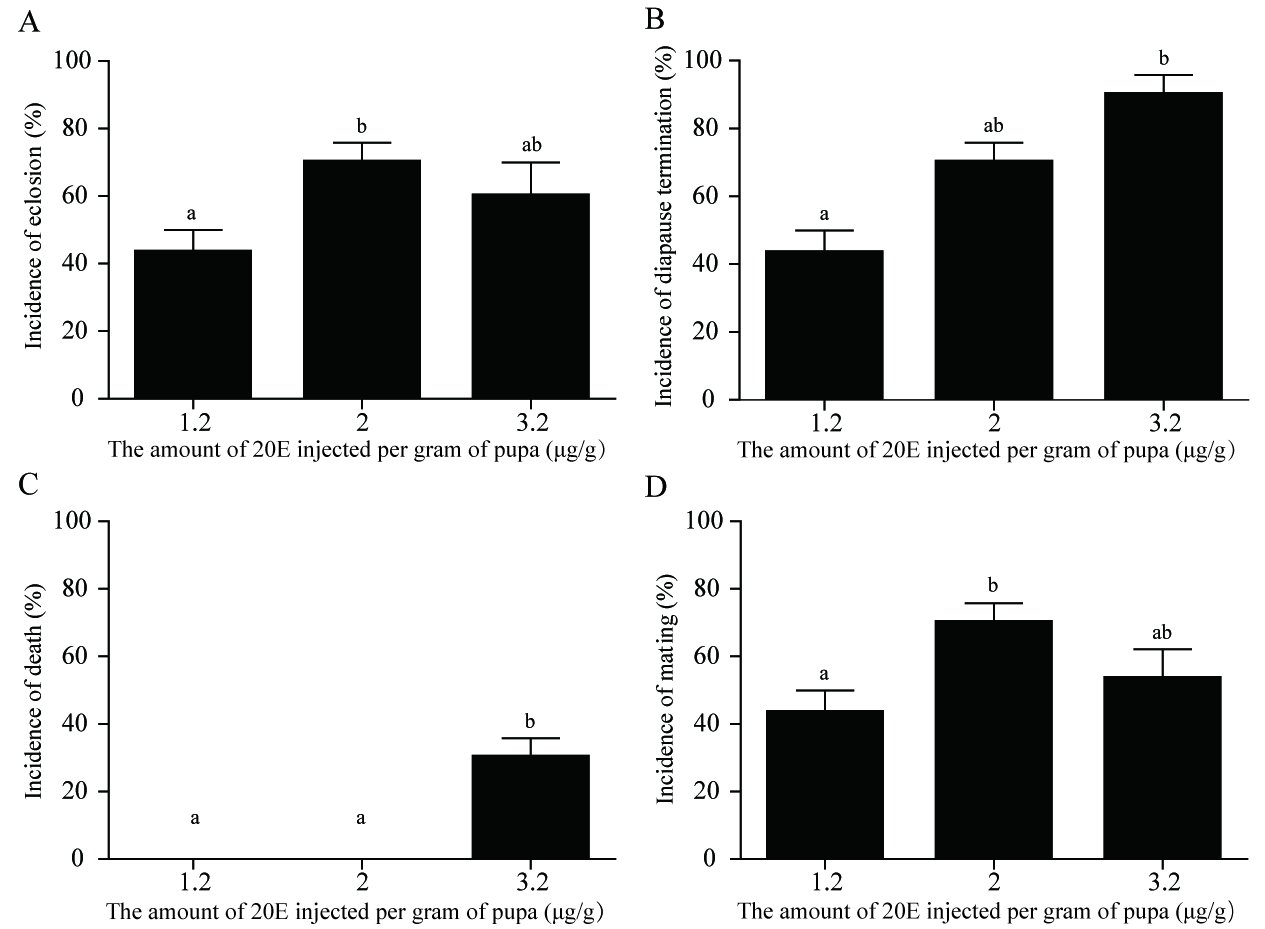

Supplement: Supplementary file 6 [file Image4.TIF]

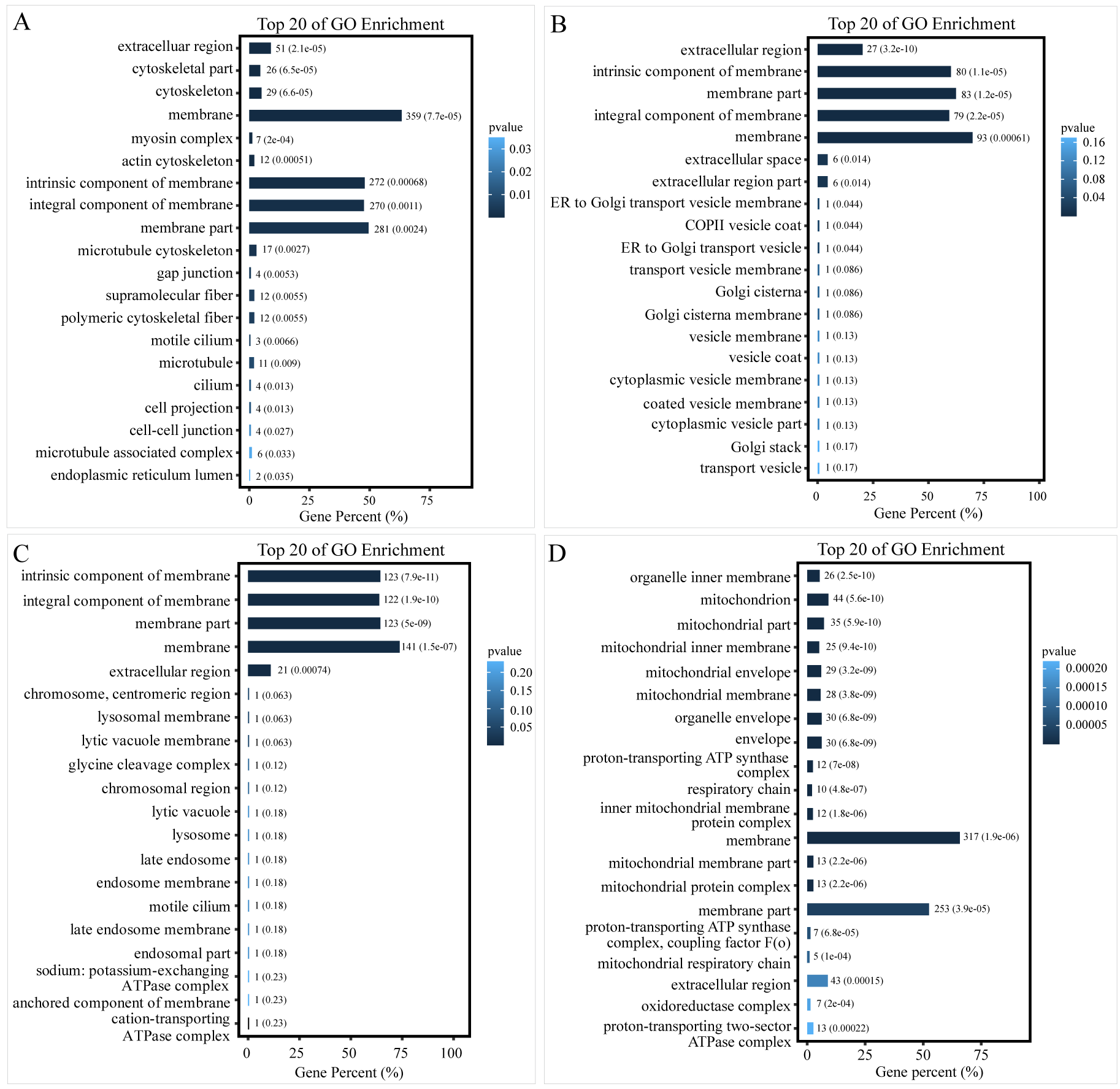

Supplement: Supplementary file 7 [file Image2.TIF]

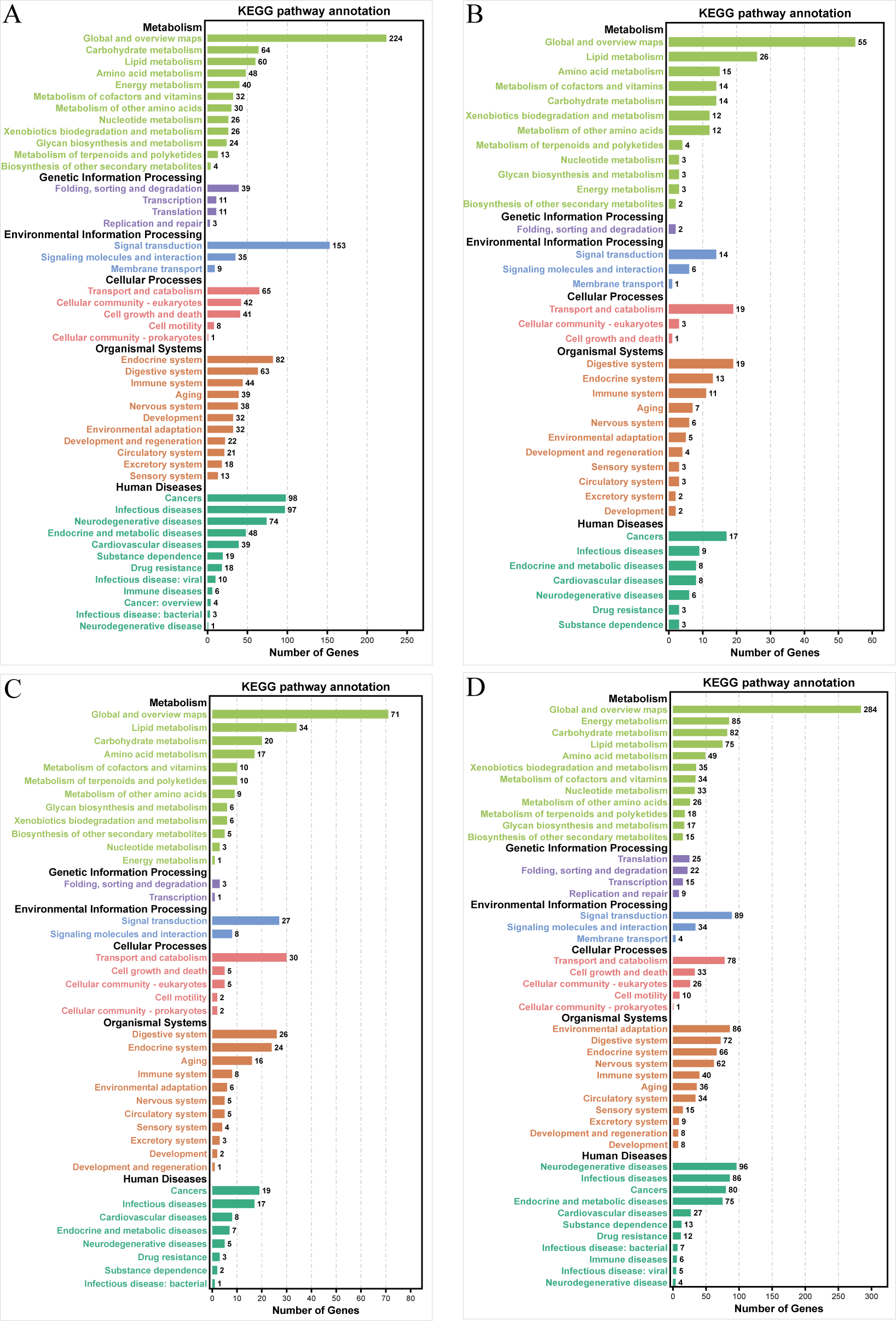

Supplement: Supplementary file 8 [file Image1.TIF]

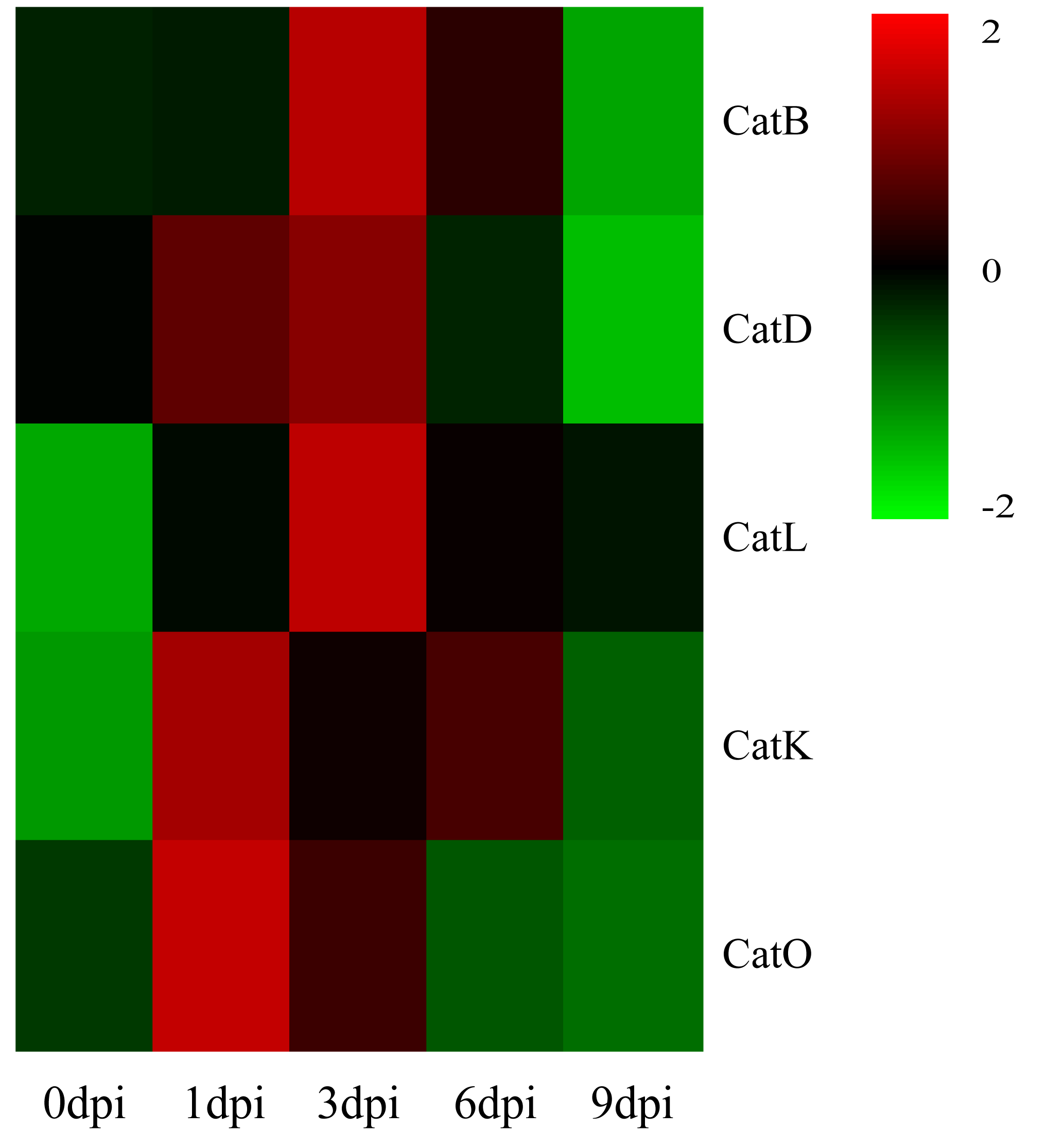

Supplement: Supplementary file 13 [file Image5.TIF]
